# 10 commandments of smile esthetics

**DOI:** 10.1590/2176-9451.19.4.136-157.sar

**Published:** 2014

**Authors:** Andre Wilson Machado

**Affiliations:** 1 Assistant Professor, Orthodontics, UFBA. Visiting Professor, MSc Program in Orthodontics, UCLA.

**Keywords:** Dental esthetics, Orthodontics, Smile

## Abstract

The search for esthetic treatment has persisted in the routine of dental professionals. Following this trend, dental patients have sought treatment with the primary aim of improving smile esthetics. The aim of this article is to present a protocol to assess patient's smile: The 10 Commandments of smile esthetics.

## Abstract (pt)

Pacientes em busca de tratamentos estéticos são uma constante na rotina de todos os profissionais que oferecem este tipo de serviço. Seguindo esta tendência, os pacientes odontológicos vêm buscando tratamentos com o objetivo primário de melhorias na estética do sorriso. O objetivo deste artigo é apresentar um protocolo de avaliação do sorriso, intitulado de "Os 10 mandamentos da estética do sorriso".

## INTRODUCTION

The search for improved dentofacial esthetics persists in modern society. Thus, inspired by pretty faces and beautiful smiles, patients have sought treatment modalities to improve dentofacial esthetics and yield positive changes in their smile.^1-6^

With a view to achieving ideal esthetic outcomes, some reference parameters must be followed. During many years, these guidelines were based on experts' opinions,^4,5,7,8,9^ in which case special attention should be given to studies conducted by Camara,^4,5^ as they provide essential information on smile esthetics. On the other hand, these clinical guidelines are questionable, since esthetics is a subjective notion and tends to vary among different individuals and cultures.^10^ This fact is a drawback for clinicians who seek a treatment protocol that involves changes in smile esthetics because many articles on this theme were based on author's opinions rather than scientific evidence.

Based on the pioneer research conducted by Kokich et al^11^, some authors sought digital imaging technology to search for more scientific and consistent references. Since then, several smile variables have been researched as follows: Smile arc;^12^ buccal corridor;^13^ amount of gingival exposure at smiling;^13,14,15^ presence of gingival and incisal asymmetry;^1,11,16,17^ presence of anterosuperior diastema;^3,14^ presence of midline shift and changes in axial proclination;^11,17^ maxillary incisors ratio, size and symmetry;^1,12^ among others.

While the wide variety of articles studying those characteristics is of paramount importance to dental literature, it hinders the work of clinicians seeking simple and practical treatment protocols. Professionals usually have a few questions: Where should smile esthetic planning begin? What are the most relevant aspects considered in esthetic treatment? Which scientific references should be considered in a given therapeutic approach?

The aim of this article is to present a protocol to assess patient's smile esthetics: "The 10 commandments of smile esthetics". It particularly aims at simplifying clinical applicability and interdisciplinary planning of smile treatment. With a view to allowing reading to flow as well as for didactic reasons, the issue discussed herein is divided into three main topics: 1) Why should smile be assessed? 2) How should smile be assessed? 3) What should be assessed - 10 commandments.

Two major aspects must be highlighted. First, interdisciplinary treatment, i.e. teamwork, is vital to yield ideal esthetic outcomes. Second, although most 10 commandments are scientific-based, treatment protocol should not be universally applied, but function as a starting point, since the concept of beauty significantly varies. Thus, all commandments presented herein must be subject to discussion among clinicians and patients so as to ensure individualized and satisfactory esthetic planning.

## 1. WHY SHOULD SMILE BE ASSESSED?

The widely known popular saying "The smile is our business card" must always be respected and considered, since there is scientific evidence evincing the smile as the most important element in the context of dentofacial esthetics.

In the last century, the scientist Alfred Yarbus^17^ designed an equipment that registered the movement of human eyes in different situations. His studies revealed that while analyzing facial photographs, people tend to focus attention mostly on the mouth and the eyes.

This hypothesis may be explained not only by the dynamic characteristic of mouth and eyes in comparison to other static structures of the face, but also by the contrast of colors: in the eye, between the iris, the pupil and the sclera; and in the mouth, between the lips, the gingival tissue, the teeth and the dark background. This finding is corroborated by recent publications confirming that during personal interactions greater attention is given to the mouth and the eyes. Additionally, because the mouth is one of the centers of attention of the face, the smile plays an essential role in facial esthetics.^18^ For this reason, we may establish the first aspect of assessing smile esthetics: the smile is a dominant component of facial esthetics.

While conducting researches at the Postgraduate Program in Orthodontics of the Federal University of Bahia (UFBA), we cast doubt on the following: Up to which point is smile really mandatory for us to assess global facial esthetics? Thus, several studies^13,15,16^ submitted manipulated images to orthodontists and laypeople who assessed them in terms of frontal view of the face and closed-up smile. Results revealed no statistically significant differences between the two assessment methods (P > 0.05). Furthermore, they demonstrated that assessment of smile esthetics in frontal view (including patient's nose, hair, eyes, facial contour, etc.) or closed-up view (highlighting patient's smile, only) yields the same degree of perception, thereby suggesting no influence of the face over esthetical assessment of different features of the smile. These data reinforce the supremacy of the smile in the context of global facial esthetics.

Once we realize the importance of the smile in a facial context, we are able to extrapolate even further. It is determining not only in the perception of facial attractiveness, but also with the perception of one's psychological characteristics. The presence or absence of deleterious alterations in an individual's smile significantly influences how this individual is perceived and evaluated.^10^ Negative alterations may affect one's personality, intelligence, emotional stability, dominance, sexuality and one's behavioral intentions of interacting with other people.^10^ These characteristics are easily perceived when dental treatment includes improvements in smile esthetics. You have certainly witnessed improvements in patient's self-esteem and quality of life after esthetic treatment is performed.

Thus, the above explains why patients seek dental treatment with chief esthetic complaint. Whenever patient's smile undergo esthetic changes they become more attractive and young with positive changes in psychological terms.

On the other hand, the issue of whether orthodontic planning has dealt with smile esthetics in order of priority is subject to discussion. The study conducted by Schabel et al,^19^ for example, revealed no strong relationship between well-finished orthodontic cases, from an occlusal standpoint, with smile esthetics. In other words, the authors suggest incorporating new criteria that includes smile esthetics in the overall evaluation of orthodontic cases.

## 2. HOW SHOULD SMILE BE ASSESSED?

Smile evaluation is basically performed by clinical means such as photographs and filming. In fact, clinical examination is prevalent in a dental context; however, registering patient's data is also necessary. To this end, photographs have always been gold standard.

Nevertheless, the validity of photographs has been recently questioned in comparison to filming used for registering one's smile. That occurs because the smile is a dynamic and complex movement comprising interaction of several facial muscles that together produce different positions of dentolabial architecture.

**Figure 1:**
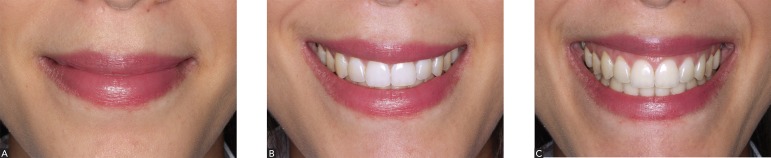
Different types of smile: **A**) commissure smile; **B**) social smile; and **C**) spontaneous smile.

According to Rubin,^21^ there are three smile levels or patterns (Fig 1). The commissure smile,^21^ also known as Mona Lisa smile, is commonly found when people greet each other in social contexts or at unusual locations such as the elevator (Fig 1A). In this smile, the commissures are pulled upward, showing or not the teeth. The second type of smile is known as cuspid^21^ or social smile. It has been globally used in self-portraits divulged in social networks. In this smile pattern, the upper lip is uniformly pulled upward showing anterosuperior teeth (Fig 1B), spontaneously or not. It oftentimes help patients with negative smile alterations (such as gingival smile) to disguise them, thereby limiting a more reliable analysis. The third smile pattern is known as complex smile^21^ characterized by movement of lower lip and wide movement of the upper lip. It is also known as spontaneous smile (usually involuntary) which realistically depicts patients' smile design (Fig 1C). According to Camara,^5^ esthetic planning should be based on complex smile, since social smile may not correspond to reality as it may represent a voluntary movement previously learned.

Thus, the difficulty in accurately registering patient's smile at the exact moment and with static photographs is clear. Furthermore, photographs are also hindered when the patient is encouraged to smile, since what is funny for some people is not funny for others.^5^

**Figure 2:**
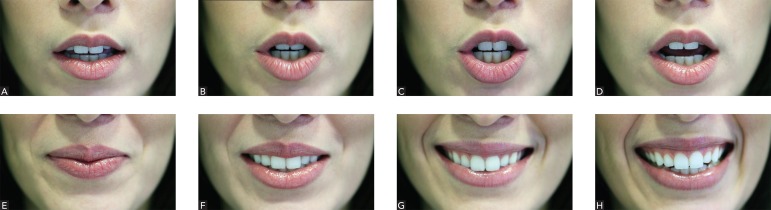
Frames showing different degrees of incisor exposure. **A-D**) at speaking and **E-H**) at smiling.

Based on the aforementioned difficulties, it seems obvious to understand that registering patient's smile by filming may provide clinicians with more reliable and elucidating data.^20^ Additionally, the same technique provides another piece of highly relevant information for esthetic treatment planning: Study of different levels of anterior teeth exposure while speaking (Figs 2A to 2D). Importantly, the filming method also has some disadvantages such as: a) The final quality of frames taken from the film is lower than the quality of photographic images; b) filming requires more data storage space (bytes); c) filming requires specific technical knowledge for taking and assessing it.^22^

In short, clinical assessment by means of through clinical examination associated with communication between clinicians and patients provides reliable data. Similarly, photographic protocols provide coherent smile data, thereby favoring esthetic treatment planning. Lastly, filming proves to be a complete and interesting tool that provides clinicians with dynamic data on smile and levels of anterior teeth exposure (Fig 2).

## 3. WHAT SHOULD BE ASSESSED - THE 10 COMMANDMENTS OF SMILE ESTHETICS

As previously mentioned, articles researching isolated features of the smile are of major scientific importance; however, they pose difficulties to clinicians who seek step-by-step instructions to plan maximum smile esthetics. Thus, this article comprises 10 topics (ten commandments) that aid, in a practical and simplified manner, orthodontic and/or esthetic planning. Furthermore, it is useful for communication between clinicians and between patients and clinicians.

The ten commandments suggested herein are as follows: 1^st^) Smile arc - Maxillary incisors in vertical position; 2^nd^) Maxillary central incisors ratio and symmetry; 3^rd^) Anterosuperior teeth ratio; 4^th^) Presence of anterosuperior space; 5^th^) Gingival design; 6^th^) Levels of gingival exposure; 7^th^) Buccal corridor; 8^th^) Midline and tooth angulation; 9^th^) Details - Tooth color and anatomical shape; 10^th^) Lip volume.

**Figure 3:**
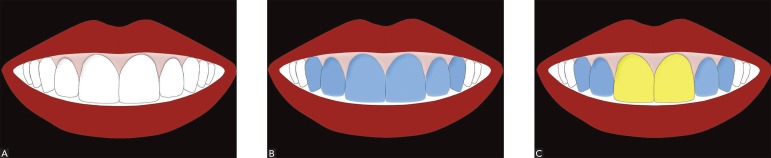
Smile illustration: **A**) ideal design; **B**) smile esthetic zone - in blue and **C**) dominance of central incisors - in yellow.

Special attention is given to disposition of anterosuperior teeth (canine to canine or first premolar to first premolar) or the area known as **esthetic zone** where central incisors are known as key elements and characterize the term "**dominance of central incisors**" (Fig 3). In short, central incisors must be highlighted as true protagonists of smile. Thus, commandments from 1 to 4 are directly related to "dominance of central incisors".

### 1^st^ commandment - Smile arc : Maxillary incisors in vertical position

Esthetic planning must begin in the noblest area of the smile: Maxillary central incisors.^7,8,9^ The 1^st^ commandment states the ideal vertical positioning for maxillary incisors at smiling. That is the first step to be planned in esthetic treatment.

**Figure 4:**
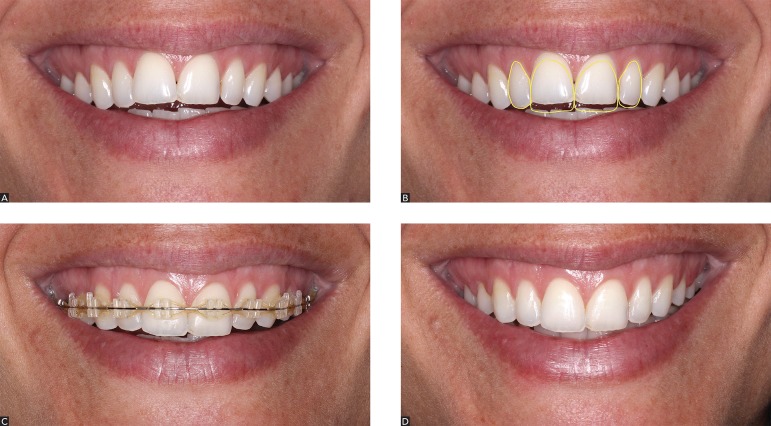
Case report showing the esthetic impact of changes in vertical positioning of incisors at smiling: **A**) initial smile showing inverted smile arc; **B**) illustration showing final incisors positioning; **C**) alignment and leveling outcomes with changes in bonding protocol following lower lip contour; and **D**) final result.

Figure 4A shows a smile with satisfactory tooth color and anatomical shape. Despite such qualities, the smile shown in Figure 4A is considered highly unesthetic, particularly due to inappropriate vertical incisors positioning considered as essential for smile esthetics.^2,8,9^

**Figure 5:**
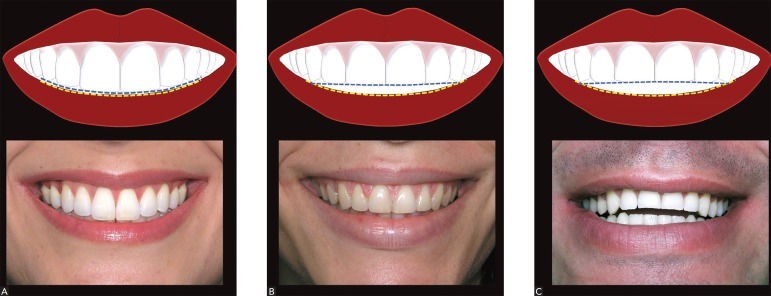
Different types of smile arc: **A**) convex or curved; **B**) plane or straight; and **C**) inverted or reverse.

An ideal smile arc has the maxillary incisal edges slightly contouring the lower lip (Fig 5A). The ideal configuration of smile arc is described as follows: convex arc, curved arc, consonant arc, deep plate-shaped arc, etc.^4,5,7,8,9,23^

On the other hand, when the incisal contour of teeth in the esthetic zone does not follow the contour of the lower lip, the smile arc is classified differently.^23^ First, it is described as plane or straight in which the incisal edges of teeth in the esthetic zone are nearly at the same level of the edges of posterior teeth, parallel to the ground and nor following the contour of the lower lip (Fig 5B). Additionally, it is also described as inverted, reverse or nonconsonant arc in which the incisal edges of teeth do not contour the lower lip and have an inverted curvature^23^ (Fig 5C).

**Figure 6:**
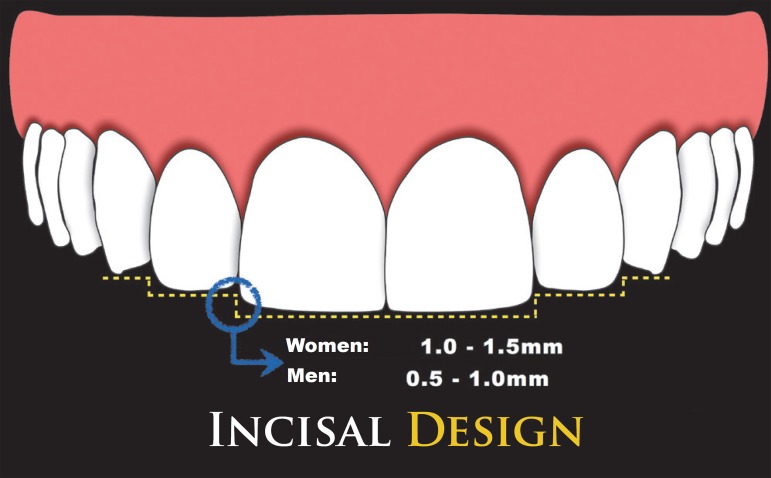
Ideal incisal contour design of teeth in the esthetic zone.

A comparison between convex and inverted smile arcs raises the following question: Why are they complete opposites from an esthetic standpoint? First, in terms of beauty of the arched contour of incisal edges of teeth in the esthetic zone, considered as the most important factor of dental esthetics (Fig 6).^9^ Second, in terms of joviality. The more arched the incisal contour of anterosuperior teeth is, the younger the smile looks; whereas the more plane, the older it looks. Additionally, according to the literature,^24^ the older someone is, the less maxillary incisor exposure and the more mandibular incisor exposure there will be both at smiling, at rest or while speaking.^24^ These changes are physiological and are caused by several factors as follows: increased perioral muscle flaccidity, genetics, ethnic group, age and sunlight exposure, all of which result in less maxillary teeth exposure.^6^

In modern society, esthetics and joviality are strongly associated, i.e., the beautiful and the young are interconnected. A few esthetic features have been highlighted in TV stars, singers and models. Greater maxillary incisor exposure at rest is one of them and has been associated with beauty, joviality, sensuality and sexuality. It is possible to infer that the current standard of beauty comprises not only a beautiful smile, but also voluminous lips and greater maxillary incisor exposure at smiling, at rest or while speaking.

**Figure 7:**
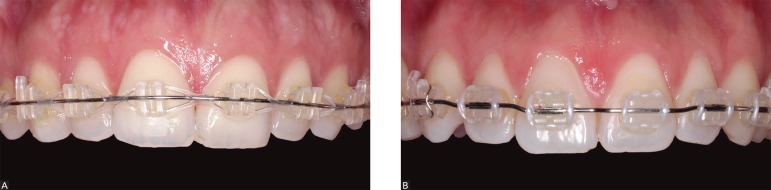
Strategies used to extrude maxillary incisors so as to achieve ideal incisal contour design and increase exposure at rest, smile and while speaking: **A**) changes in height of bracket positioning; and **B**) orthodontic arch bends.

This finding may guide the following dental planning modalities: Esthetic restoration and/or rehabilitation, manufacture of complete denture and vertical movement of incisors during orthodontic treatment. In orthodontic treatment, the clinician may adapt the protocol of bracket bonding and/or add bends to orthodontic archwires with a view to increasing incisors extrusion and, therefore, rendering them more visible at rest and at smiling by means of achieving proper smile arc (Fig 7).

We conducted another research to test the vertical position of maxillary central incisors and found that slightly extruded central incisors were more attracted than slightly intruded ones. Results reveal that the vertical position of incisors is when the edge of central incisors is near the lower lip and far from the incisal edge of lateral incisors and canines, thereby ensuring dominance of central incisors.^2^ In other words, the incisal edge of maxillary central incisors must be below the cuspid tip of canines (Fig 5A).

With a view to aiding clinicians to achieve ideal design of incisal contour in the esthetic zone, the step between central and lateral incisors must range from 1.0 - 1.5 mm for women and 0.5 - 1.0 mm for men (Fig 6).^2^ This finding reveals that convex smile arcs are more suitable for women (Fig 5A) whereas convex or plane arcs are acceptable for men (Fig 5B).

After discussing this concept, we are able to reassess Figure 4A, in which case the need for maxillary central incisor extrusion to fulfill the 1^st^ commandment is clear (Fig 4B). Importantly, the need for individualizing orthodontic bracket bonding should be highlighted. Should height guidance provided by the brackets manufacturer had been used in this clinical case, suggesting that canines should be as high as central incisors, treatment would hardly achieve the ideal smile arc. It would achieve a plane arc instead. Similarly, should bonding be based on brackets positioned on the center of clinical crowns, the ideal curved smile arc would not be achieved. Thus, orthodontic bonding should be individualized in the esthetic zone, following patient's lower lip contour and anatomical shape of teeth. Figure 4C shows bracket positioning following this principle and with the major aim of extruding central incisors. In this case, the height of brackets bonded to canines was 3.5 mm, whereas the height of brackets bonded to central incisors was 5.5 mm. Thus, after alignment and leveling, maxillary central incisors were ideally positioned in accordance with the aforementioned recommendations, thereby achieving a pleasant and young smile (Fig 4D).

Importantly, planning vertical changes of teeth in the esthetic zone requires that three important points be considered: The first regards occlusal maxillary plane and head inclination while assessing patient's smile. Clockwise maxillary plane and head inclination lead to greater incisor exposure. As a result, convex smile arcs are more easily found. On the other hand, counterclockwise maxillary occlusal plane inclination and patient's head inclination backwards hinder convex smile arcs to be seen and/or achieved.

The second point is with regards to mandibular function which must be absolute in dental planning. In other words, esthetic goals must not disrupt occlusal balance. Incisor extrusion or intrusion may influence protrusion and laterality. Therefore, mandibular function must be carefully assessed in which case occlusal adjustments might render necessary.^2^

The third point to be considered is axial proclination of maxillary and mandibular incisors (interincisal angle). This feature is a determining factor that allows or not incisors extrusion, thereby increasing smile visibility at rest and while speaking. In the event of proclined incisors (decreased interincisal angle), extrusion is hindered or hampered as in cases of Class I bimaxillary protrusion or Class I division I malocclusion. In these cases, incisors angulation must be corrected so as to optimize vertical positioning.

To bring this commandment to a conclusion, we carefully reassess Figure 6 which shows that, with a view to ideally adjusting the incisal contour of teeth in the esthetic zone, gingival margin positioning also changes. In most clinical cases, clinicians face the following: If central incisors incisal edge is below canines incisal edge, what is the final design of gingival margins? Such questioning is answered by the 5^th^ commandment.

**Summary of the 1^st^ commandment**

» Vertical positioning of maxillary incisors is determining to achieve an attractive, young smile.

» The incisal edge of maxillary central incisors must be bellow the cuspid tip of canines, ensuring dominance of central incisors.

» The step between central and lateral incisors must range from 1.0 to 1.5 mm for women and from 0.5 to 1.0 mm for men.

### 2^nd^ commandment - Ratio and symmetry of maxillary central incisors

**Figure 8:**
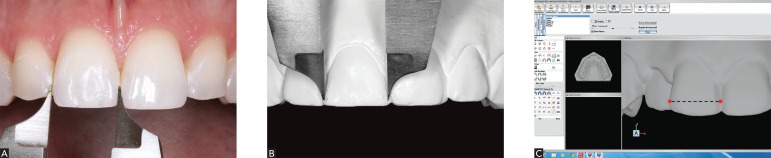
Different methods employed to measure maxillary central incisors width and height: **A**) clinical caliper measurement; **B**) caliper measurement in conventional model; and **C**) software measurement in digital model.

**Figure 9:**
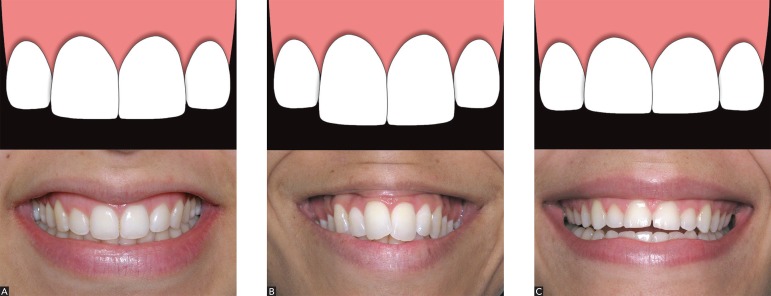
Different width-height ratio of central incisors: **A**) ideal ratio, between 75 and 85%; **B**) long teeth with ratio < 75%; and **C**) short or squared teeth with ratio > 85%

Once maxillary incisors vertical positioning is determined, maxillary central incisors ratio and symmetry are adjusted. Thus, the 2^nd^ commandment asserts that ideal width-height (W/H) ratio and symmetry of central incisors must be achieved. The clinician must register the width and height of maxillary central incisors clinical crowns so as to determine W/H ratio (Fig 8). Subsequently, he must plan 75 to 85% ratios which are considered more esthetic (Fig 9A).^25^ Should values tend towards 75%, central incisors will have a longer pattern widely accepted by women, whereas in 85% ratios, incisors will have a wider pattern widely accepted by men.

In the event of altered W/H ratios, the first step consists in determining whether one of the central incisors has proper W/H ratio. Should that be the case, this tooth will be used as reference (template) to change the other central incisor. Should both central incisors be altered, their height is used as reference for correction. In other words, esthetic central incisors usually have 9.5 to 11-mm high crowns.^8,9^

**Figure 10:**
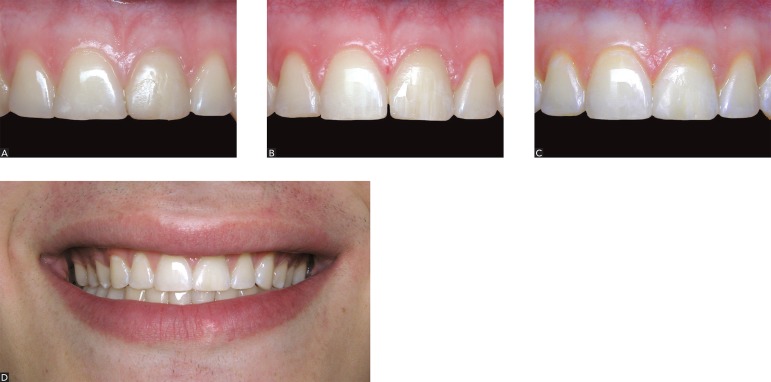
Clinical case illustrating the importance of W/H ratio in smile esthetics: **A**) initial closed-up view of maxillary incisors; **B**) after orthodontic appliance removal; **C**) final result; **D**) final smile.

Figure 10 shows a patient whose chief complaint was having a big tooth in the esthetic zone. His right central incisor was 9.1-mm wide and 9.5-mm high, thereby producing a W/H ratio of 95%, highly unesthetic. His left central incisor, however, was 8.0-mm wide and 9.5-mm high, thereby producing a W/H ratio of 84% which is within normality. Thus, treatment comprised 0.5-mm interproximal wear on the mesial and distal surfaces of right central incisor, followed by orthodontic space closure. As a result, ideal W/H ratio remained on the left side, whereas it changed on the right side. Subsequently, with a view to fulfilling the 2^nd^ commandment, left central incisor reconstruction was repeated so as to achieve maximum symmetry between central incisors.

**Figure 11:**
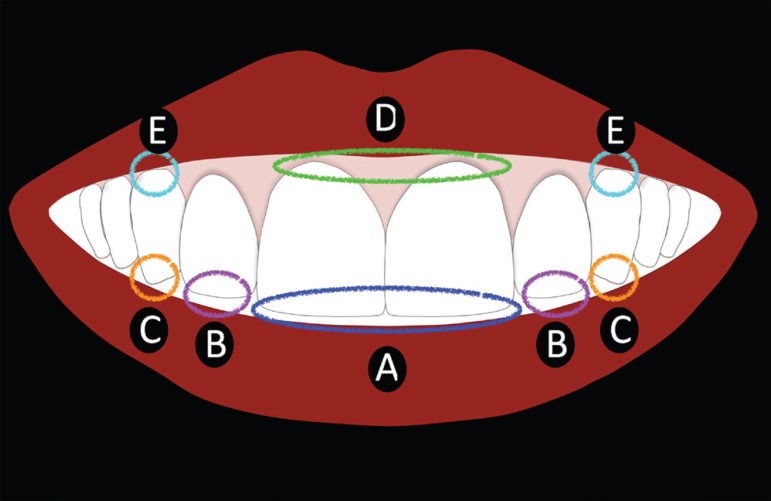
Limits of esthetic acceptability of incisal and gingival asymmetry in the esthetic zone: **A**) 0.5 mm^1^; **B**) 1.0 mm^1^; **C**) 2.0 mm^17^; **D**) from 1.5 to 2.0 mm^11,14^; and **E**) from 1.5 to 2.0 mm^16^.

The demand for symmetry between central incisors is based on the clinical assumption that the nearer the midline, the greater the need for symmetry, and the further from the midline, the higher the number of slight asymmetries clinically acceptable.^9^ With a view to testing this hypothesis, we conducted a research assessing the esthetic impact of central and lateral incisor asymmetries on the smile of two adult female patients (Caucasian and melanoderma).^1^ Our results corroborate the aforementioned hypothesis, since a slight 0.5-mm maxillary central incisor asymmetry was identified as unesthetic by orthodontists and laypeople. On the other hand, slight asymmetries on the side of incisors may go unnoticed,^1^ while in canines, even greater asymmetries may not be identified (Fig 11).^16^

**Figure 12:**
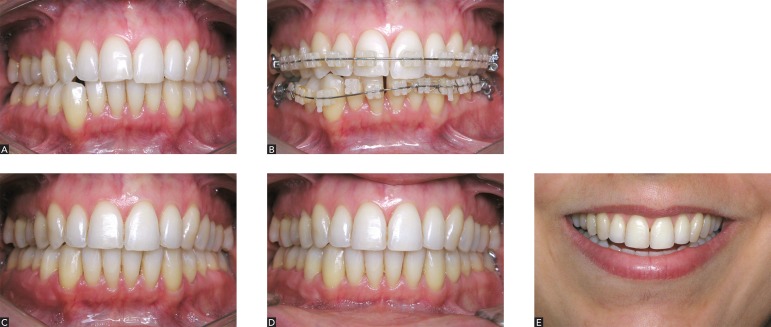
Clinical case illustrating the importance of symmetry between central incisors in smile esthetics: **A**) initial; **B**) during orthodontic treatment; **C**) after orthodontic appliance removal; **D**) final result; and **E**) final smile.

Hence, in cases requiring orthodontic finishing, we suggest that multidisciplinary treatment be conducted to achieve maximum symmetry between maxillary central incisors. Figure 12, for instance, shows left central incisor W/H ratio of 78% used as template for treatment. After orthodontic treatment, the patient was referred to cosmetic restoration of right central incisor and reshaping so as to fulfill the 2^nd^ commandment, thereby achieving proper W/H ratio and maximum symmetry between maxillary central incisors.

**Summary of the 2^nd^ commandment**

» Take note of width/height ratios for maxillary central incisors.

» Aim at esthetic proportion (75 - 85%) and maximal symmetry.

» Symmetry between incisal edges is the most important aspect.

### 3^rd^ commandment - Proportion between anterosuperior teeth

**Figure 13:**
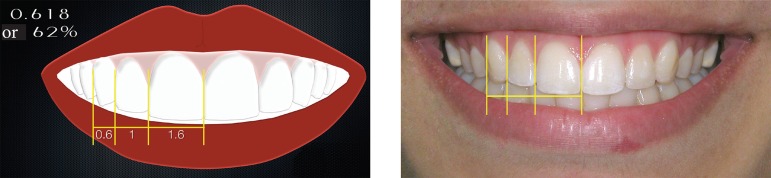
Smile with golden ratio (62%) between teeth in the esthetic zone.

Once the ideal vertical positioning of maxillary incisors is achieved and W/H ratio as well as maximum symmetry between central incisors is attained, the proportion between anterosuperior teeth is then adjusted. This feature is widely considered in Dentistry and it is based on the golden ratio initially proposed by Levin in 1978.^26^ According to the author, in frontal view, there exists a width proportion of teeth seen in perspective. This fact is shown by Figure 13 in which visible lateral incisor width accounts for 62% of central incisor width, while canine width accounts for 62% of lateral incisor width.

A recently published research^27^ compared several different proportions, such as 57% (featuring narrower lateral incisors), 67%, 70% and 72% (featuring wider lateral incisors).^27^ Results revealed that the golden ratio should be applied with caution, as the value of 62% must be interpreted as a mean rather than a standard to be pursued. Furthermore, greater proportions (67% and 70%) have been highlighted as being more esthetic, thereby revealing that there seems to exist a strong preference for wider instead of narrower incisors.

**Figure 14:**
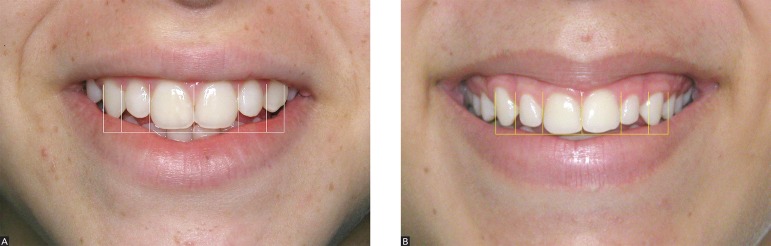
Digital ratio grid used with two narrow smiles: **A**) golden ratio grid (62%) and **B**) grid with modified ratio (70%).

Clinically, this feature is easily noticeable in view of conoid or extremely narrow lateral incisors. There are reference rulers and guides used in the clinical practice. Additionally, digital symmetry guides or grids are very useful tools that respect standard proportions and allow us to study and visualize this variable on computer and/or tablet screens. Figure 14, for instance, shows two grids, one used with golden ratio (62% - Fig 14A) and another one used with modified proportion (70% - Fig 14B). They demonstrate that in both smiles, lateral incisors are narrow and do not respect the most esthetically pleasant proportion between anterosuperior teeth.

**Figure 15:**
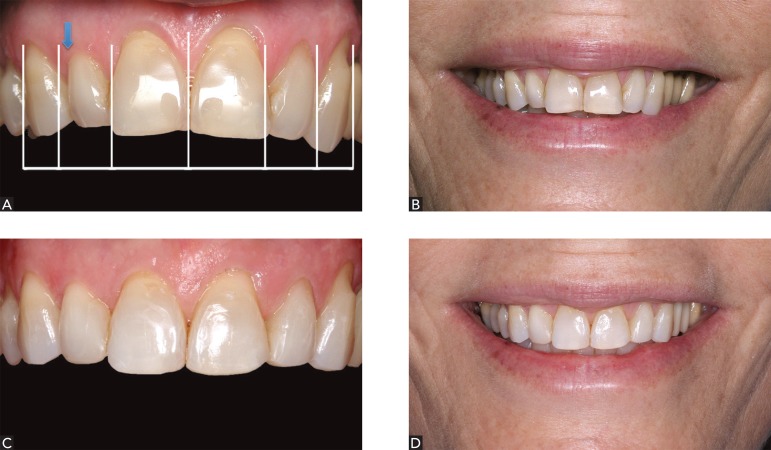
Clinical case illustrating the importance of proportion between anterosuperior teeth: **A**) initial closed-up view in the esthetic zone showing right lateral incisor of reduced width (blue arrow); **B**) initial smile; **C**) final result showing adequate proportion between teeth in the esthetic zone; **D**) final smile.

The case described in Figure 15 shows asymmetrical proportion between anterosuperior teeth. The golden ratio grid makes it easier to clearly identify the discrepancy, revealing that the right lateral incisor had reduced mesiodistal dimension. Orthodontic treatment opened up a space in the lateral incisor area which would undergo further esthetic restoration so as to fulfill the 3^rd^ commandment which is the proportion between anterosuperior teeth. Furthermore, reshaping was performed to improve symmetry between central incisors and adjust the step between central and lateral incisors, emphasizing the dominance of central incisors in one's smile.

**Summary of the 3^rd^ commandment**

» Too narrow lateral incisors are unesthetic.

» Multidisciplinary treatment is necessary.

### 4^th^ commandment - Presence of anterosuperior spaces

Esthetic perception of diastema in the esthetic zone is widely discussed in the literature. At the same time, it arouses considerable controversy. Nevertheless, one should question the following: Are diastemas in the esthetic zone esthetic or unesthetic? According to the literature,^14^ small midline diastemas (not greater than 2.0 mm) might go unnoticed by laypeople. This finding may somehow explain why some famous artists have diastemas and find such spaces attractive. On the other hand, this finding^14^ might also be questioned, since it is too optimistic in terms of the impact midline diastemas have over smile esthetics. Do 1.0-2.0 mm diastemas really go unnoticed by laypeople?

**Figure 16:**
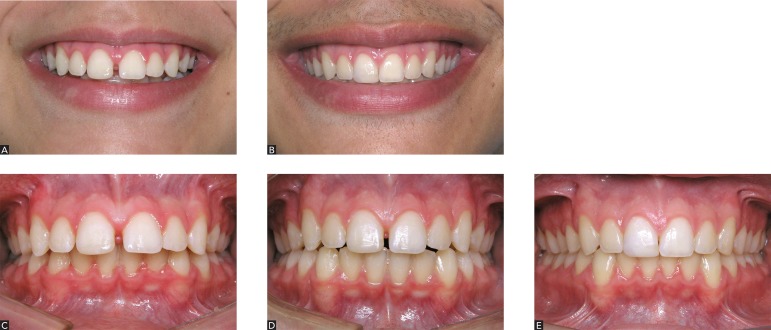
The impact of midline diastema correction over patient's smile: **A**) initial smile; **B**) final smile; **C**) initial frontal view; **D**) frontal view after activator use; **E**) final frontal view after fixed corrective orthodontic treatment.

**Figure 17:**
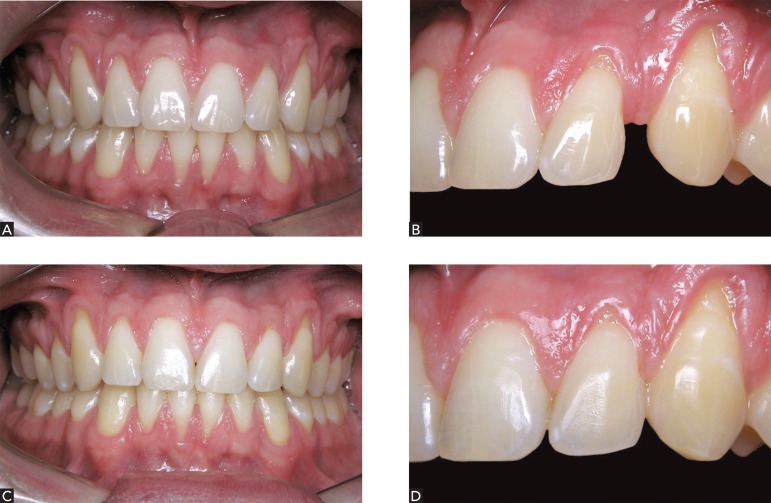
Diastema correction in maxillary lateral incisors area: **A**) initial frontal view; **B**) initial lateral view of maxillary left incisor; **C**) final frontal view; and **D**) final view in maxillary lateral incisors area.

Although esthetics is highly subjective, the 4^th^ commandment asserts that all midline diastemas must be closed either by orthodontic or multidisciplinary treatment. One should also ask whether diastema in the lateral incisors area (mesial, distal or both) affects smile esthetics. With a view to answering this question, we conducted another research to assess the esthetic impact of diastemas over two female patients' smile.^3^ Results revealed that the greater the diastema and the nearer the midline, the more unesthetic the smile is. The only exception was for 0.5-mm diastemas in the distal surface of lateral incisors, which were not identified by laypeople. Thus, if space is to remain after orthodontic treatment, the distal surface of lateral incisors should be the area of choice.^3^ Figures 16 and 17 show two cases of diastemas in the esthetic zone. In the former, the remaining space was between central incisors; whereas in the latter, the remaining space was in the distal surface of the left lateral incisor. In both cases, with a view to fulfilling the 4^th^ commandment, all remaining spaces were closed.

**Summary of the 4^th^ commandment**

» Diastemas in the esthetic zone are unesthetic.

» All diastemas should be closed.

### 5^th^ commandment - Gingival design

**Figure 18:**
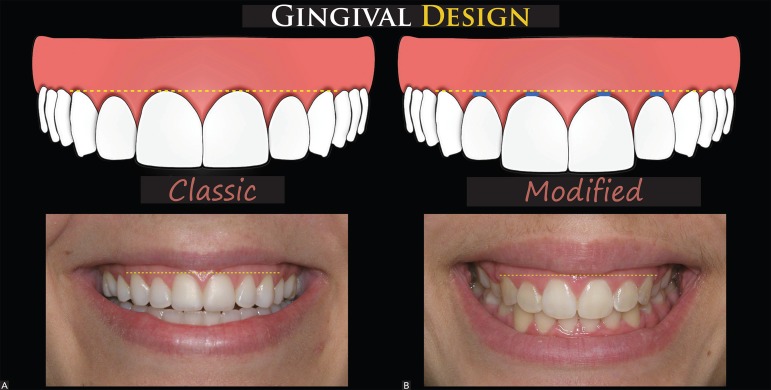
Two different gingival margin designs: **A**) Classic: leveled canine and central incisor margins, with lateral incisor margin slightly below; **B**) Modified: central incisor margin below canines and lateral incisor margins leveled with central incisors or slightly below them.

Gingival tissue architecture must also be taken into account in esthetic treatment. The terms "pink esthetics" and "red esthetics" have been used to describe ideal gingival contour at smiling. Some dental textbooks bring the following parameter of ideal esthetic gingiva: "Canine gingival margin must coincide with central incisors gingival margin, whereas lateral incisors gingival margin must be slightly below this line" (Fig 18A). Indeed, such parameter provides maximum smile esthetics. However, should clinicians follow the aforementioned parameter in cases in which canines and central incisors are equal in length,^2^ they might position central incisors incisal edge at the same level or above canines. As a result, plane or inverted smile arcs might be produced, and so are unesthetic smiles.

This clinical doubt arouses from the following: Which esthetic parameter is more important? Incisal contour (white esthetics) or gingival design (pink esthetics)? We have recently published a research in which we establish a relationship between esthetic perception and incisal edge smile line as well as gingival margin smile line.^2^ Results reveal that incisal smile design (white esthetics) is the most important factor of dental esthetics.^9^ Thus, in addition to what is recommended in the 1^st^ commandment (smile arc), one may opt for a modified gingival design in which the gingival margin of central and lateral incisors coincide and are slightly (0.5 - 1.0 mm) below canines, the gingival margin of central incisors is below canines (0.5 - 1.0 mm) and the gingival margin of lateral incisors is below central incisors (0.5 mm) (Fig 18B). It is clear that extrusion of central incisors must be conducted according to patient's lower lip contour and sex, respecting the recommendation of greater extrusion of incisors for female smiles. Furthermore, the degree of extrusion must not violate lateral guidance.^2^

Another esthetic parameter widely divulged is the positioning of gingival apexes defined as the most apical points of gingival contour. Frontal analysis of teeth in the esthetic zone reveals that gingival apexes are located in the center of the crowns or slightly distally. On the other hand, based on the limits of acceptability of smile asymmetry (Fig 11), changes in gingival apexes hardly affect one's smile negatively.

Importantly, even after determining the ideal design of gingival margins in the esthetic zone, the clinician might face gingival asymmetry between teeth. Asymmetry between incisal edges of central incisors are considered unesthetic.^1^ But how about gingival asymmetry? Can it be identified by laypeople? According to the literature, gingival asymmetry not greater than 1.5 - 2.0 mm between central incisors^11,14^ go unnoticed by laypeople. We conducted another research at the Federal University of Bahia (UFBA) to investigate the esthetic impact of gingival asymmetry between canines^16^ and found the same limit of perception (1.5 - 2.0 mm) for laypeople. These findings highlight once again that white esthetics is more important than pink esthetics (Fig 11).

**Figure 19:**
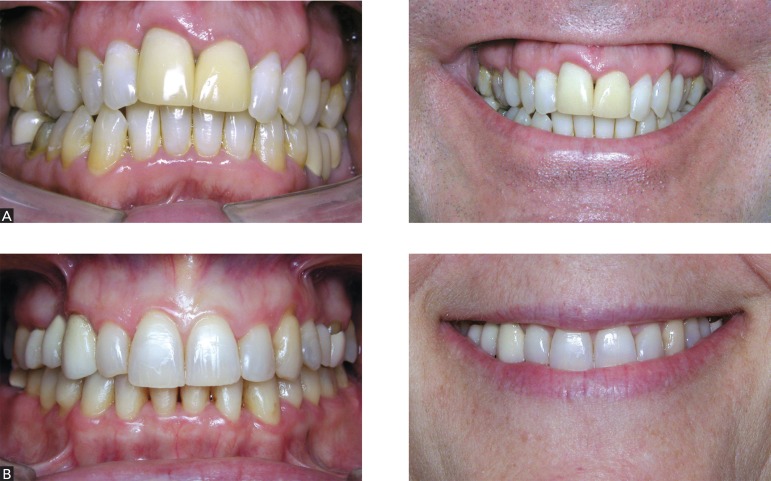
Two clinical cases with gingival asymmetry in the esthetic zone requiring different treatment procedures: **A**) real need for intervention due to great gingival asymmetry exposure at smiling; and **B**) smile without gingival asymmetry exposure and with no need for treatment.

Even though a number of studies yields positive results regarding the esthetic impact of asymmetry,^11,14^ the 5^th^ commandment asserts that after determining ideal gingival design, whether classic or modified, the clinician should focus on correcting potential asymmetries, provided that they are evident at smiling. Gingival smile displays greater asymmetry and, for this reason, must be corrected. Nevertheless, little gingival display at smiling does not require correction (Fig 19). It is worth noting that should discrepancies be visible at smiling and near the midline, the need for correction if even greater.

**Figure 20:**
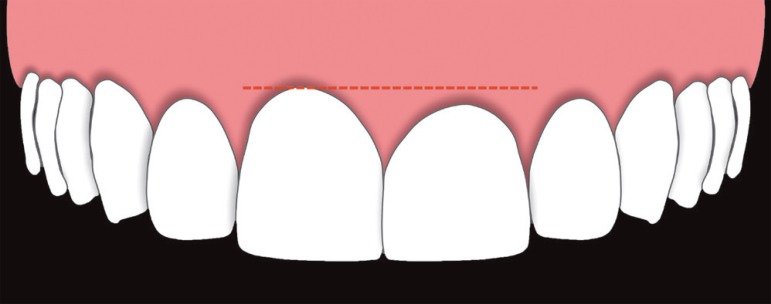
Gingival asymmetry between maxillary central incisors.

Cases of gingival discrepancy between central incisors (Fig 20) are basically corrected by either one of the following three treatment methods: a) gingivoplasty of the lowest incisor; b) intrusion and incisal restoration of one central incisor; c) extrusion of one central incisor with posterior incisal wear.^28^ The first step to choose the ideal treatment option is to apply the 2^nd^ commandment (maxillary central incisors proportion and symmetry) and determine which central incisor is gold standard. In this case, it is tooth #11, which requires gingivoplasty (a) or intrusion (b). Subsequently, treatment planning requires that the cementoenamel junction be identified by means of clinical probing and periapical radiograph or tomography so as to determine whether gingivoplasty is feasible or not.

**Figure 21:**
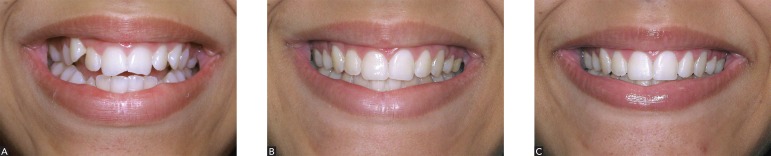
Clinical case illustrating incisal and gingival asymmetry correction: **A**) initial smile; **B**) orthodontic treatment result; and **C**) final smile.

Figure 21 shows a patient with improved smile esthetics after removal of fixed orthodontic appliances; however, with discrepancy between gingival margins and incisal edges of central incisors. Treatment comprised gingivoplasty of right central incisor and esthetic reconstruction of left central incisor, thereby fulfilling all aforementioned commandments.

**Summary of the 5^th^ commandment**

» Gingival margin of central incisors must be leveled or slightly bellow (0.5 to 1.0 mm) canines.

» Gingival margin of lateral incisors must be leveled or slightly bellow (0.5 mm) central incisors.

» Multidisciplinary treatment is necessary for ideal gingival design adjustment

### 6^th^ commandment - Gingival exposure

**Figure 22:**
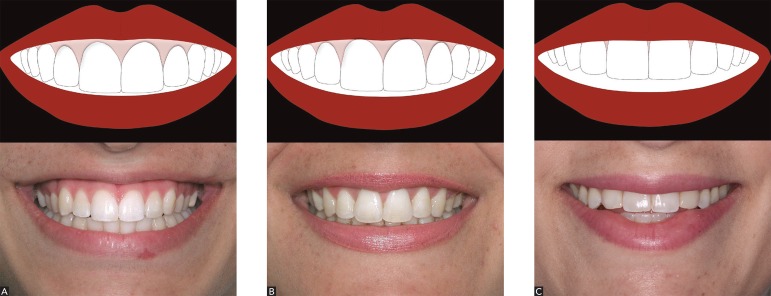
Different smile lines according to Tjan et al.^23^ **A**) high smile, characterized by total exposure of clinical crowns and continuous strip of gingival tissue; **B**) medium smile, characterized by great (75%) or total (100%) exposure of clinical crowns and interdental or interproximal papillae; **C**) low smile, characterized by clinical crown exposure not greater than 75% and no gingival tissue.

Assessing the amount of teeth and gingival tissue exposure in the esthetic zone is of paramount importance for smile esthetics. According to Tjan et al,^23^ gingival exposure is determined by the smile line classified as high, medium or low (Fig 22).

Importantly, the ideal smile does not require gingival tissue exposure to be eliminated. In fact, many TV stars, models and role models of beauty display the entire length of teeth and little gingival tissue at smiling. As previously mentioned, greater exposure of incisors and little gingival exposure at smiling are esthetic and characteristic of joviality. The major point of clinical scientific discussion is as follows: Is gingival tissue exposure at smiling esthetic? If so, what is the ideal amount of gingival exposure? To what extent is gingival exposure acceptable?

According to the literature, gingival tissue exposure at smiling is not a negative feature.^11,14,15^ In a previous study,^15^ we found that the maximum limit of gingival tissue exposure is of 3.0 mm, thereby corroborating other studies.^11,14^ Thus, gingival exposure not greater than 3.0 mm is perfectly acceptable, whereas values greater than 3.0 mm are considered unesthetic. Based on these findings and considering the different types of smile (high, medium and low, as shown in Fig 22), the 6^th^ commandment suggests that high smile with gingival exposure not greater than 3.0 mm is more esthetic, followed by medium and low smiles.

Since the theme of gingival smile has already been widely reviewed, it will not be brought to discussion in this manuscript. For this reason, we recommend further reading on the topic.^29^

The two major aspects to be discussed on the theme of gingival exposure are: a) The need for a treatment planning that contemplates the primary etiology of the case and, therefore, avoids potential risk of failure; b) Avoiding intrusion of maxillary incisors by complying with the aforementioned points. A very common clinical mistake consists in intruding maxillary incisors so as to minimize gingival exposure in cases of normal smile arc. In these cases, loss of ideal incisal smile contour (1^st^ commandment) might be more deleterious than gingival tissue exposure.

**Figure 23:**
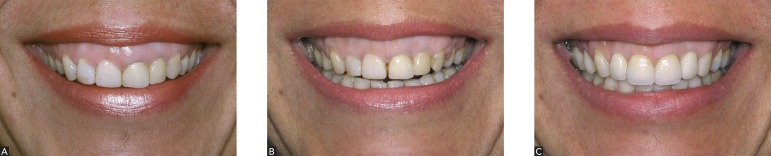
Clinical case illustrating gingival smile treatment: **A**) initial smile; **B**) orthodontic treatment outcome, illustrating ideal incisal design; and **C**) final smile.

Figure 23 shows a smile with great gingival tissue exposure. Orthodontic treatment was performed with extraction of first premolars and, after removing the fixed appliances, the patient was referred to gingivoplasty and manufacture of dental veneers in the esthetic zone. Subsequently, with the aid of dermatological procedures^5^ that included the use of botulinum toxin, gingival tissue exposure was minimized, thereby favoring satisfactory esthetic outcomes. Importantly, despite being a case of gingival smile, the 1^st^ commandment was fulfilled with an ideal smile arc as well as proper design of incisal edges and modified gingival design.

**Summary of the 6^th^ commandment**

» Gingival exposure not greater than 3 mm is not unesthetic.

» Intrusion of maxillary incisors, especially central incisors, should be avoided.

» Gingival smile treatment should be guided by its etiology.

### 7^th^ commandment - Buccal corridor

**Figure 24:**
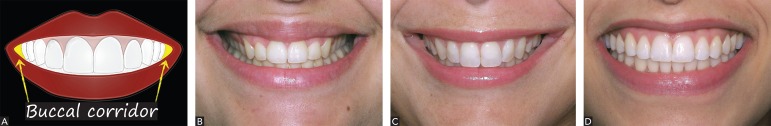
Different types of buccal corridor: **A**) buccal corridor at smiling; **B**) wide buccal corridor; **C**) intermediate buccal corridor; and **D**) narrow buccal corridor.

Buccal corridor is the bilateral space between the vestibular surface of visible maxillary posterior teeth and lip commissure at smiling (Fig 24A).^8,9^ Basically, there are three types of buccal corridors: a) wide, usually followed by narrow maxillary dental arch (Fig 24B); b) intermediate, followed by dental arches of intermediate transverse dimensions (Fig 24C); and c) narrow or nonexistent, associated with severe transverse dental arches (Fig 24D). Literature does not present a consensus regarding the esthetic impact of buccal corridor over smiling. While some studies demonstrate that different buccal corridors do not affect smile esthetics, other state the opposite. We conducted another research at the Federal University of Bahia^13^ and found intermediate buccal corridors to be more esthetic in comparison to wide and narrow buccal corridors. Following this trend, wider buccal corridors are more unesthetic.^12,13^

Indeed, when this feature is compared to all the aforementioned ones, we come to the conclusion that one's buccal corridor is not as critical to smile esthetics. In spite of that, the 7^th^ commandment suggests that intermediate buccal corridors are ideal, followed by narrow or nonexistent ones. Thus, cases of wide buccal corridors require rapid maxillary expansion and/or dental expansion so as to enhance smile esthetics.

**Summary of the 7^th^ commandment**

» Buccal corridor is not critical in smile esthetics.

» Intermediate buccal corridor is more attractive, whereas wide buccal corridor (narrow smile arch) is more unesthetic.

» Wide buccal corridor should be avoided and maxillary expansion should be indicated whenever necessary.

### 8^th^ commandment - Midline and tooth angulation

Similarly to the buccal corridor, midline deviation plays a controversial role in smile esthetics. However, it is hardly noticed by laypeople. According to the literature, midline deviations not greater than 3-4 mm are not identified by laypeople.^11,14^ This explains why even though some famous artists and models have severe midline deviation, they are still considered as role models of beauty.

**Figure 25:**
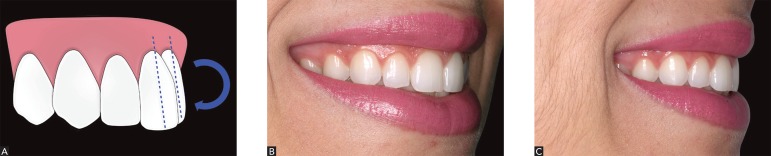
Importance of assessing incisor angulation in lateral view: **A**) ideal torque; **B**) oblique radiograph; and **C**) perpendicular radiograph.

While midline deviations are hardly noticed by laypeople, changes in tooth angulation in the esthetic zone (alone or in combination) are extremely deleterious to one's smile. According to the literature,^14^ minimal changes of 2.0 mm in angulation of anterior teeth in frontal view are considered unesthetic by laypeople. For this reason, they must be corrected. Correction of angular discrepancies must be based on classic esthetic literature guidance: The incisal edge line of central incisors must be parallel to the interpupillary line.^7,8,9^ Additionally, incisor torque, especially central incisors, must change in lateral smile view, given that from this point of view, smile esthetics is analyzed differently in comparison to frontal view (Fig 25). Thus, changes in incisor angulation must be investigated from frontal as well as lateral smile view.

**Figure 26:**
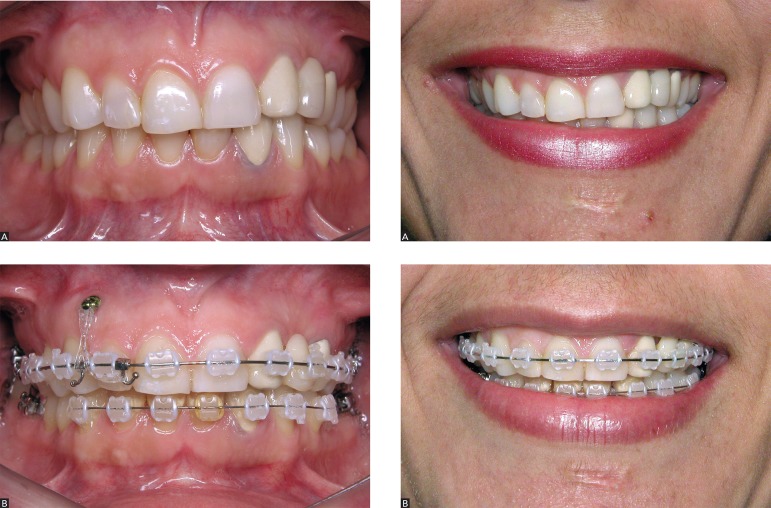
Clinical case illustrating the negative impact of changes in incisor angulation in frontal view: **A**) initial frontal photograph and smile photograph; **B**) intermediate result after incisal plane and angulation correction with the aid of mini-implant.

Figure 26 shows a case with both problems: Midline deviation and changes in tooth angulation in the esthetic zone. One can easily notice that correcting the second is prioritized over the first. Mini-implant was used to correct changes in tooth angulation. Additionally, once parallelism between the incisal edge line of central incisors and interpupillary line was restored, esthetic benefits were evinced.

Although the literature determines a limit of perception of 3-4 mm for laypeople to identify midline deviation and 2.0 mm to identify changes in tooth angulation, the 8^th^ commandment suggests that midline deviations equal to or greater than 2.0 mm and any degree of changes in tooth angulation must be corrected.

**Summary of the 8^th^ commandment**

» Midline deviation is less relevant than changes in tooth angulation in the esthetic zone.

» Midline deviation equal to or greater than 2.0 mm and any degree of changes in tooth angulation must be corrected.

### 9^th^ commandment - Tooth color and anatomical shape

Procedures comprising this commandment are usually performed in the orthodontic finishing phase. The 9^th^ commandment basically determines three procedures to aid esthetic refinement: a) Dental bleaching; b) Adjustment of contacts; c) Reshaping of incisal edges in the esthetic zone.

**Figure 27:**
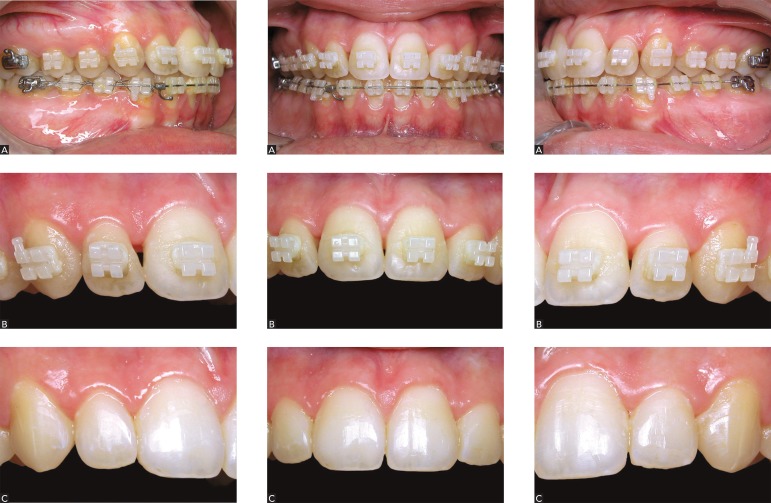
Clinical case illustrating the importance of detailing and tooth anatomical shape: **A**) orthodontic finishing phase; **B**) closed-up view of the esthetic zone showing black triangles caused by inappropriate contact; **C**) final results after teeth reshaping.

**Figure 28:**
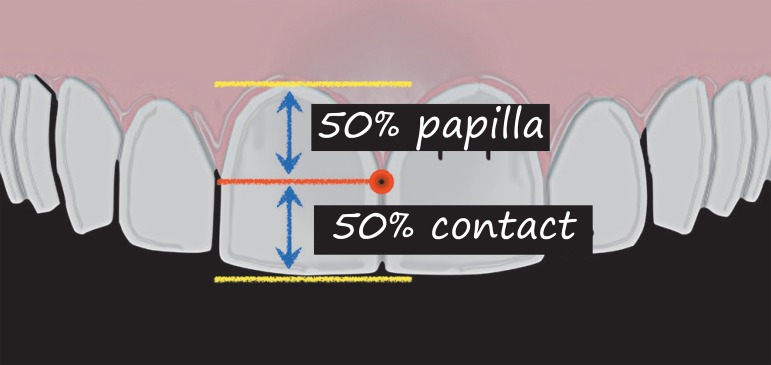
Diagram illustrating the ideal position of contact between central incisors so as to favor filling of interproximal spaces by interdental papillae.

**Figure 29:**
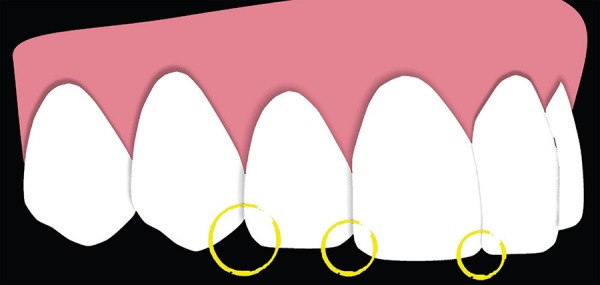
Diagram illustrating the ideal disposition of incisal embrasures, showing a natural and progressive increase from central incisors to canines.

Figure 27A shows a case of orthodontic finishing. This example casts doubt on the following: What is missing in this case? A closed-up view of teeth in the esthetic zone reveals the presence of black triangles and absence of papillae in interproximal spaces (Fig 27B). Papillae must fill interdental spaces up to the contacts. However, when contacts are inappropriate, interdental spaces might remain. Papilla/contact relationship in central incisors is of 1:1, for this reason, interdental space is half occupied by the papilla and half occupied by contact (Fig 28).^28^ Thus, interproximal wear was carried out to position the contact in the mid portion of clinical crowns, thereby favoring closing of black triangles and ideally filling the papillae (Fig 27C). With a view to enhancing incisal edges contour, slight wear was also carried out to minimize incisal embrasures, thus improving esthetics and giving the smile a younger look (Figs 27C and 29).

**Summary of the 9^th^ commandment**

» Dental bleaching is highly indicated to improve final results.

» Contact adjustments are necessary to correct potential black spaces'.

» Enameloplasty by means of enamel wear or veneer placement to enhance incisal design esthetics.

### 10^th^ commandment - Lip volume

The last commandment is related to the structure that frames the smile: the lips. The current standard of beauty comprises not only a beautiful smile, but also voluminous lips and greater maxillary incisor exposure at smiling, at rest or while speaking.

**Figure 30:**
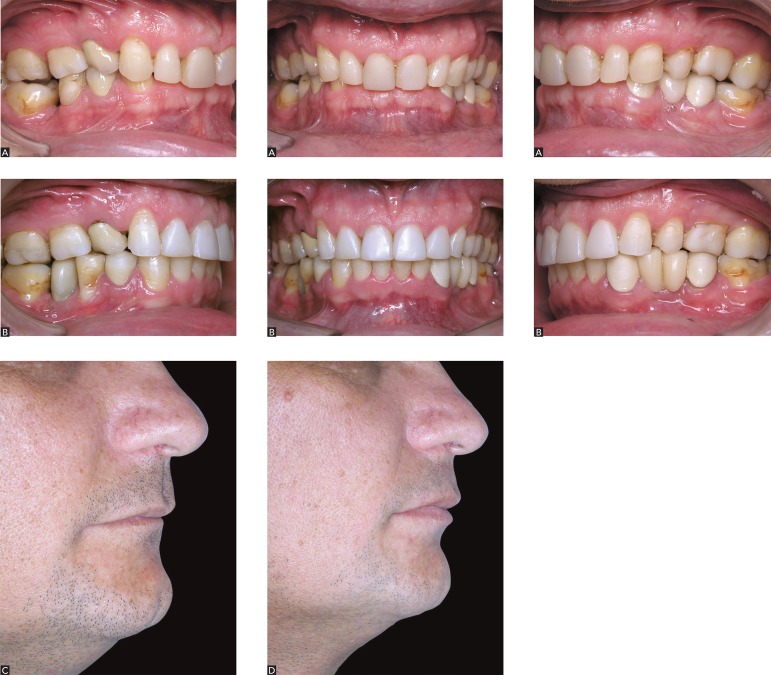
Case report illustrating increased lip volume after orthodontic treatment: **A**) initial; **B**) final; **C**) initial profile showing thin lips; and **D**) final profile showing increased lip volume.

**Figure 31:**
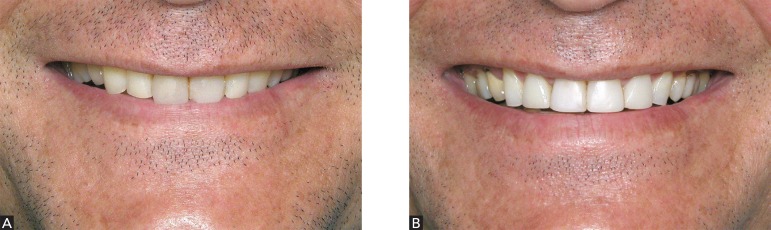
Clinical case shown in Figure 30: **A**) initial smile; and **B**) final smile.

According to the literature, anteroposterior positioning of teeth plays a key role in determining lip volume.^5,30^ As an example, Figures 30 and 31 show a 38-year-old patient with deep bite and severe reduction in lip vermilion exposure. Once deep bite and incisor proclination (particularly of lower incisors) were corrected, there were significant improvements in lip volume, thereby enhancing lip esthetics and giving the patient a younger look. Importantly, in spite of severe deep bite, intrusion of maxillary teeth was not carried out to prevent smile aging. Teeth retraction must be carefully considered, since lip volume may decrease, thereby resulting in thinner unesthetic lips.

Orthodontists may also recommend multidisciplinary treatment carried out by means of filling agents for lip augmentation. This theme has been extensively discussed and, for this reason, we recommend further reading on the topic.^5,30^

**Figure 32:**
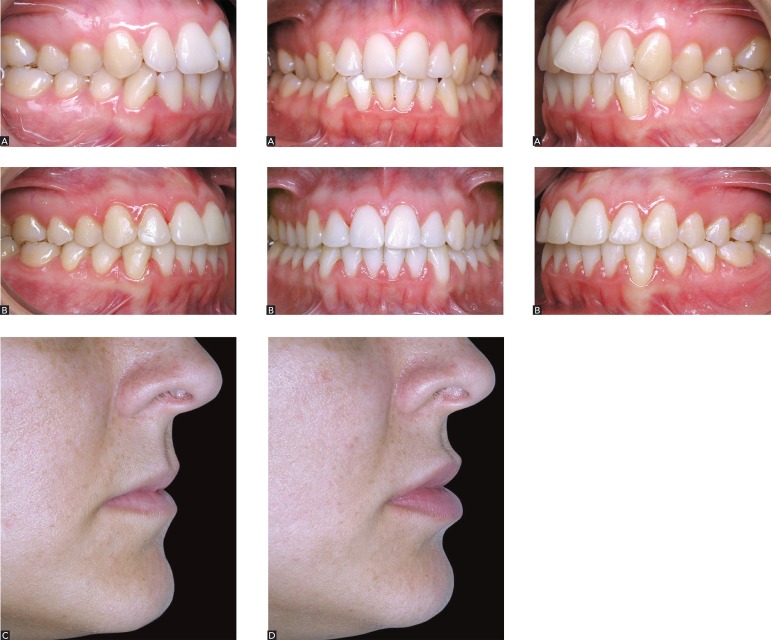
Clinical case illustrating multidisciplinary treatment associating Orthodontics and Dermatology to correct smile and lip volume: **A**) initial; **B**) final; **C**) initial profile showing thin lips; and **D**) final profile showing increased lip volume after using filling agents for lip augmentation.

**Figure 33:**
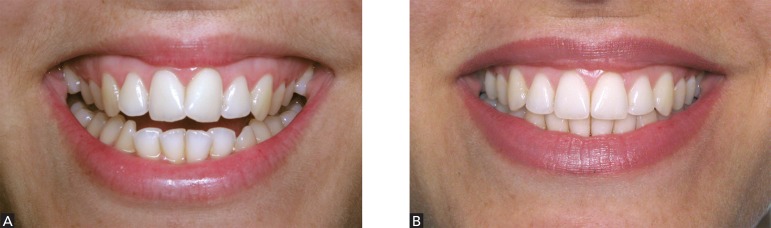
Clinical case shown in Figure 32: **A**) initial smile; and **B**) final smile showing esthetic benefits produced by multidisciplinary treatment.

As an example of filling agents for lip augmentation, Figures 32 and 33 show a case of mild crowding with unpleasant smile and thin lips. Orthodontic treatment and dermatological procedures carried out by means of filling with hyaluronic acid yielded satisfactory results with a pleasant smile and greater lip volume, thereby fulfilling the 10^th^ commandment (lip volume).

**Summary of the 10^th^ commandment**

» Voluminous lips are the current standard of beauty.

» Care should be taken when performing retraction of anterior teeth so as to prevent negative impact over lip volume.

» Multidisciplinary treatment associating Dentistry and Dermatology is necessary for lip filling, when needed.

## FINAL CONSIDERATIONS

The 10 Commandments of smile esthetics may be considered a starting point for clinicians who aim at achieving maximum esthetic in dental treatment. Special attention should be given to the first four commandments associated with dominance of central incisors at smiling.

Importantly, treatment should be discussed with patients so as to individualize treatment planning and, as a result, fulfill their desires. Lastly, interdisciplinary treatment, i.e. teamwork, is vital to yield ideal esthetic outcomes.
